# Long-Term prognosis of radiofrequency catheter ablation for atrial fibrillation with different subtypes of heart failure in the era of ablation index guidance

**DOI:** 10.3389/fcvm.2022.922910

**Published:** 2022-09-20

**Authors:** Yu Qiao, Zhen Zhao, Xiang Cai, Yulong Guo, Mingpeng Fu, Ke Liu, Jinrui Guo, Tao Guo, Guodong Niu

**Affiliations:** ^1^Department of Cardiac Arrhythmia, Fuwai Yunnan Cardiovascular Hospital, Kunming Medical University, Kunming, China; ^2^State Key Laboratory of Cardiovascular Disease, Cardiac Arrhythmia Center, Fuwai Hospital, National Center for Cardiovascular Diseases, Chinese Academy of Medical Sciences and Peking Union Medical College, Beijing, China

**Keywords:** atrial fibrillation, heart failure, catheter ablation, ablation index, long-term outcome

## Abstract

**Background:**

The long-term outcomes of ablation index (AI)-guided radiofrequency catheter ablation (RFCA) on atrial fibrillation (AF) and different subtypes of heart failure (HF) remain unknown. The aim of the study was to evaluate the long-term prognosis of AI-guided RFCA procedures in patients with AF and concomitant HF.

**Methods:**

We retrospectively included consecutive patients with AF and HF who underwent the initial RFCA procedure with AI guidance from March 2018 to June 2021 in our institution. The patients were categorized into two groups: HF with preserved ejection fraction (HFpEF) group and HF with mid-range ejection fraction (HFmrEF) +HF with reduced ejection fraction (HFrEF) group.

**Results:**

A total of 101 patients were included. HFpEF and HFmrEF + HFrEF groups consisted of 71 (70.3%) and 30 patients (29.7%), respectively. During a median follow-up of 32.0 (18.2, 37.6) months, no significant difference was detected in AF recurrence between groups (21.1 *vs*. 33.3%) after multiple procedures, whereas the incidence of the composite endpoint of all-cause death, thromboembolic events, and HF hospitalization was significantly lower in HFpEF group (9.9 *vs*. 25.0%, Log-rank *p* = 0.018). In multivariable analysis, a history of hypertension [hazard ratio (*HR*) 4.667, 95% confidence interval (*CI*) 1.433–15.203, *p* = 0.011], left ventricular ejection fraction (LVEF) < 50% (*HR* 5.390, 95% *CI* 1.911–15.203, *p* = 0.001) and recurrent AF after multiple procedures (*HR* 7.542, 95% *CI* 2.355–24.148, *p* = 0.001) were independently associated with the incidence of the composite endpoint.

**Conclusion:**

Long-term success could be achieved in 75% of patients with AF and concomitant HF after AI-guided RFCA procedures, irrespective of different HF subtypes. Preserved LVEF was associated with a reduction in the composite endpoint compared with impaired LVEF. Patients with recurrent AF tend to have a poorer prognosis.

## Introduction

Atrial fibrillation (AF) and heart failure (HF) frequently coexist (1, 2). The presence of AF has been demonstrated to worsen the prognosis of HF, with increased risk of stroke, HF hospitalization and death, and vice versa (3–6). Based on left ventricular ejection fraction (LVEF), HF could be divided into three distinct subtypes: HF with preserved ejection fraction (HFpEF), HF with mid-range ejection fraction (HFmrEF), and HF with reduced ejection fraction (HFrEF) (5).

Radiofrequency catheter ablation (RFCA) is a well-established therapy for AF (7) not only in patients with HF (8), but mostly in normal hearts (9). Recent evidence has suggested that RFCA should improve the symptom and prognosis of AF with HF, with significant reduction in all-cause mortality and HF hospitalization compared with medical therapy (8, 10). At the same time, AF may be precipitated by secondary causes such as hypertension, hyperthyroidism (11), alcohol consumption (12), smoking (13), and finally channelopathies (14, 15).

The ablation index (AI), a novel parameter formulated with contact force (CF), ablation duration and power, has been shown to be associated with lesion transmurality and durability in RFCA procedures (16, 17). Moreover, AI-guided ablation strategy has been demonstrated to improve both procedural and long-term outcomes (18–20). Consequently, it is more and more widely adopted in AF ablation. However, the long-Term prognosis of AI-guided RFCA on AF and different subtypes of HF remains unknown.

The purpose of the present study was to evaluate the long-term outcomes of AI-guided RFCA procedures in patients with AF and concomitant HF based on different subtypes.

## Methods

### Study population

This retrospective observational cohort study included consecutive patients with AF and concomitant HF who underwent the initial RFCA procedures from March 2018 to June 2021 in Fuwai Yunnan Cardiovascular Hospital. Patients with New York Heart Association (NYHA) class I or IV, acute decompensated HF or cardiogenic shock, severe valvular disease (defined as a history of aortic or mitral replacement or repair, evidence of severe aortic or mitral regurgitation, severe aortic stenosis, or moderate to severe mitral stenosis), congenital heart disease, or hypertrophic obstructive cardiomyopathy were not eligible. Patients with LVEF ≥ 50% were defined as HFpEF, those with an LVEF of 40–49% were defined as HFmrEF, and those with LVEF < 40% were defined as HFrEF. Finally, patients were categorized into two groups: HFpEF group and HFmrEF + HFrEF group (combined because of the small number of patients in each group). The informed consent on receiving RFCA was obtained from all patients. This study complies with the Declaration of Helsinki and was approved by the local ethics committee.

### Radiofrequency catheter ablation procedure

The RFCA procedures were performed under conscious sedation. The details of the procedure have been described previously (21). After access to the left atrium (LA) *via* the transseptal puncture needle (Synaptic Medical), two 8.5—French sheaths (NaviEase, Synaptic Medical) were advanced into LA. Intravenous heparin was administered immediately after the transseptal puncture and to maintain the activated clotting time of 250–350 s throughout the procedure. A 7-French mapping catheter (PentaRay, Biosense Webster) was used to perform the 3-dimensional electrical anatomical mapping (EAM) under the guidance of the CARTO3 system (Biosense Webster). An 8-French 3.5-mm tip irrigated ablation catheter (SmartTouch, Biosense Webster) with an upper power of 40 W, an upper temperature limit of 43°C, and a flow rate of 17 ml/min was used to perform the CF-guided ablation. Real-time radiofrequency (RF) applications were visualized using CARTO VISITAG Module with predefined settings of catheter stability (2.5 mm for 5 s) and minimum CF of 5 g. RF energy was delivered with AI of 400 at the posterior wall/roof/SVC and AI of 450 at the anterior/inferior wall/mitral isthmus line/CTI. Inter-lesion distance between two neighboring lesions was controlled within 5 mm. If any RF application delivered at each site did not fulfill the predefined target AI values, a new RF delivery would be applied at the same site until reaching the target values.

The stepwise ablation strategy was employed: (i) circumferential pulmonary vein isolation (CPVI) with bi-directional electric block as the endpoint in every patient; in case of pulmonary vein (PV) reconnection in redo procedures, PV reisolation with gaps ablation was performed. (ii) if AF remained after CPVI, the complex fractionated atrial electrogram (CFAE)-based ablation was performed, with the above-mentioned location-specified AI as the target of each ablation application. (iii) if focal or re-entrant atrial tachycardia (AT) was presented during the procedure, a focal or linear ablation based on the EAM and/or entrained mapping was performed. (iv) if cavo-tricuspid isthmus (CTI) dependent atrial flutter (AFL) was observed before or during the procedure, linear ablation of the CTI was performed with di-directional conduction block as the endpoint. (v) if AF persisted after all these ablation lesions, intravenous ibutilide and/or electric cardioversion was used to restore sinus rhythm.

### Follow-up

All patients were monitored for 48 h in the hospital after the RFCA procedure and treated with dabigatran or rivaroxaban for at least 3 months. Antiarrhythmic drugs were continued for 3 months after the procedure and was then stopped if no AF recurrence was found.

After the 3-month blanking period, subsequent follow-up consisted of a clinical interview, electrocardiograms (ECGs), and 24-h Holter monitoring every 3 months for 1 year, and then every 6 months. In addition, the ECGs were recorded at the time of symptoms. Early AF recurrence was defined as symptomatic and/or asymptomatic episodes of AF/AFL/AT lasting > 30 s identified on the 12-lead surface electrocardiogram or Holter monitoring within the 3-month of blanking period, while AF recurrence was defined as these episodes after the blanking period.

### Endpoint definition

The composite endpoint was defined as a composite of all-cause death, thromboembolic (TE) events and HF hospitalization. The NYHA class, N-terminal pro-B type natriuretic peptide (NT-ProBNP) level, and TTE parameters such as left atrial diameter (LAD), left ventricular end-diastolic diameter (LVEDD), and LVEF at baseline and the end of follow-up were also analyzed.

### Statistical analysis

Continuous variables with normal distribution were described as the mean ± *SD* for normally distributed data, and comparisons between groups were performed with Student's *t*–test. Non-normally distributed continuous data were summarized as median (interquartile range) and compared with the Mann–Whitney test. Categorical variables were described as counts and compared by the chi-square test. Survival curves were generated with the Kaplan–Meier analysis and compared by the log-rank test. Cox regression analysis was used to determine the independent predictors for AF recurrence and the composite endpoint, with a determination of hazard ratio (*HR*) and 95% confidence interval (*CI*) for each variable in the model. Variables selected for testing in the multivariate analysis were those with *p* < 0.05 in the univariate model.

All tests were 2-tailed, and a statistical significance was established at *p* < 0.05. All analyses were performed using R 4.0.4 and SPSS software (version 22.0; SPSS, Inc., Chicago).

## Results

### The baseline characteristics of the study population

**Figure 1:**
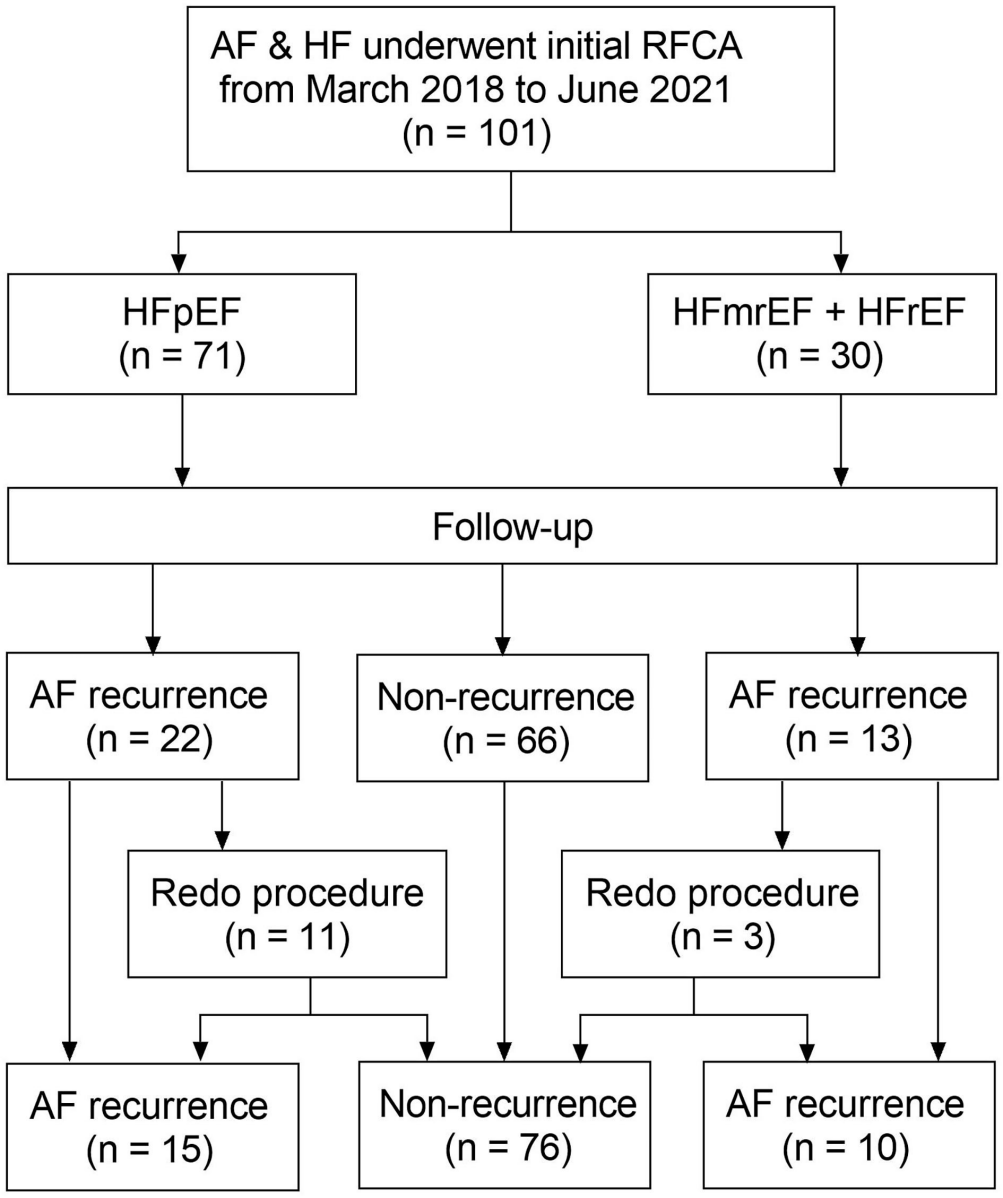
Flow diagram of the study. AF, atrial fibrillation; HF, heart failure; RFCA, radiofrequency catheter ablation; HFpEF, heart failure with preserved ejection fraction; HFmrEF, heart failure with mid-range ejection fraction + heart failure; HFrEF, heart failure with reduced ejection fraction.

A flow diagram of the present study is presented in Figure 1. A total of 101 patients (mean age 63.3 ± 11.5 years) with AF and concomitant HF underwent the initial RFCA procedure were included. HFpEF and HFmrEF + HFrEF groups consisted of 71 (70.3%) and 30 patients (29.7%), respectively. The baseline characteristics of the study population are presented in Table 1. Compared with HFpEF group, patients in HFmrEF + HFrEF group were younger (57.2 ± 12.2 *vs*. 65.8 ± 10.2, *p* < 0.001), had lower CHA_2_DS_2_-VASc scores [2.5 (1.0, 3.0) *vs*. 3.0 (2.0, 4.0), *p* = 0.005], a higher prevalence of dilated cardiomyopathy (36.7 *vs*. 14.1%, *p* = 0.011), higher estimated glomerular filtration rates (77.3 ± 23.9 *vs*. 67.5 ± 21.4, *p* = 0.045), serum uric acid levels (478.8 ± 135.9 *vs*. 410.4 ± 97.1, *p* = 0.005), and larger LVEDDs (56.4 ± 6.7 *vs*. 46.0 ± 4.2, *p* < 0.001).

**Table 1:** The baseline characteristics of the study population.

**Variables**	**Overall *n* = 101**	**HFpEF *n* = 71**	**HFmrEF + HFrEF *n* = 30**	***P*-value**
**Demographic data**				
Age, years	63.3 ± 11.5	65.8 ± 10.2	57.2 ± 12.2	< 0.001
Male sex, *n (%)*	61 (60.4)	34 (47.9)	17 (56.7)	0.556
BMI, kg/m^2^	24.4 ± 3.4	24.6 ± 3.1	24.0 ± 4.0	0.352
Non-paroxysmal AF, *n (%)*	55 (54.5)	36 (50.7)	19 (63.3)	0.344
AF duration, month	12.0 (2.0, 36.0)	12.0 (2.0, 36.0)	11.0 (1.0, 36.0)	0.717
CHA_2_DS_2_-VASc score	3.0 (2.0, 4.0)	3.0 (2.0, 4.0)	2.5 (1.0, 3.0)	0.005
**NYHA class**				
Class II	83 (82.2)	61 (85.9)	22 (73.3)	0.131
Class III	18 (17.8)	10 (14.1)	8 (26.7)	0.131
**HF etiology**				
DCM, *n (%)*	21 (20.8)	10 (14.1)	11 (36.7)	0.011
HCM, *n (%)*	3 (3.0)	3 (4.2)	0 (0)	0.255
IHD, *n (%)*	19 (18.8)	16 (22.5)	3 (10.0)	0.141
TIC, *n (%)*	36 (35.6)	23 (32.4)	13 (43.3)	0.294
Undefined, *n (%)*	23 (22.8)	20 (28.2)	3 (10.0)	0.047
**Past medical history**				
Hypertension, *n (%)*	58 (57.4)	42 (59.2)	16 (53.3)	0.749
Diabetes mellitus, *n (%)*	16 (15.8)	14 (19.7)	2 (6.7)	0.179
CHD, *n (%)*	25 (24.8)	22 (31.0)	3 (10.0)	0.048
Stroke, *n (%)*	12 (11.9)	11 (15.5)	1 (3.3)	0.165
**Medication**				
beta-blocker, *n (%)*	57 (56.4)	40 (56.3)	17 (56.7)	1.000
ACEI/ARB/ARNI, *n (%)*	24 (23.8)	15 (21.1)	9 (30.0)	0.483
MRA, *n (%)*	22 (21.8)	10 (14.1)	12 (40.0)	0.009
Diuretics, *n (%)*	12 (11.9)	1 (1.4)	11 (36.7)	< 0.001
Anticoagulant, *n (%)*	16 (15.8)	11 (15.5)	5 (16.7)	1.000
Anti-platelet, *n (%)*	12 (11.9)	12 (16.9)	0 (0.0)	0.039
Statin, *n (%)*	30 (29.7)	27 (38.0)	3 (10.0)	0.010
AADs, *n (%)*	18 (17.8)	14 (19.7)	4 (13.3)	0.630
**Laboratory findings**				
NT-ProBNP, pg/ml	865.4 (369.7, 1784.0)	859.2 (317.4, 1852.5)	871.5 (625.2, 1661.5)	0.640
SUA, umol/L	430.7 ± 113.8	410.4 ± 97.1	478.8 ± 135.9	0.005
eGFR, ml/min/1.73 m^2^	70.4 ± 22.5	67.5 ± 21.4	77.3 ± 23.9	0.045
**TTE parameter**				
LAD, mm	41.9 ± 6.3	41.2 ± 6.5	43.6 ± 5.6	0.070
LVEDD, mm	49.1 ± 6.9	46.0 ± 4.2	56.4 ± 6.7	< 0.001
LVEF, %	56.5 ± 11.9	63.2 ± 6.2	40.6 ± 5.0	< 0.001

HFpEF, heart failure with preserved ejection fraction; HFmrEF, heart failure with mid-range ejection fraction; HFrEF, heart failure with reduced ejection fraction; BMI, body mass index; AF, atrial fibrillation; NYHA, New York Heart Association; HF, heart failure; DCM, dilated cardiomyopathy; HCM, hypertrophic cardiomyopathy; IHD, ischemic heart disease; TIC, tachycardia-induced cardiomyopathy; CHD, coronary heart disease; ACEI, angiotensin-converting enzyme inhibitor; ARB, angiotensin receptor blocker; ARNI, angiotensin receptor neprilysin inhibitor; MRA, mineralocorticoid receptor antagonist; AAD, anti-arrhythmic drug; NT-ProBNP, N-terminal pro-B type natriuretic peptide; SUA, serum uric acid; eGFR, estimated glomerular filtration rate; TTE, transthoracic echocardiography; LAD, left atrial diameter; LVEDD, left ventricular end-diastolic diameter; LVEF, left ventricular ejection fraction.

### Procedural data

The ablation details in the initial and redo procedures are presented in Table 2. In the initial procedures, CPVI was achieved in all patients. There was no significant difference in the ablation of anterior wall line, CFAE and CTI between the two groups. However, ablation at the mitral isthmus line (26.7 *vs*. 8.5%, *p* = 0.035), roof line (40.0 *vs*. 16.9%, *p* = 0.013), and SVC (30.0 *vs*. 9.9%, *p* = 0.025) was more frequently performed in patients with HFmrHF + HFrEF compared with those with HFpEF.

**Table 2:** The ablation details of the initial and redo procedures.

**Ablation lesion**	**Initial procedure**			**Redo procedure**		
	**HFpEF *n* = 71**	**HFmrEF + HFrEF *n* = 30**	***P*-value**	**HFpEF *n* = 11**	**HFmrEF + HFrEF *n* = 3**	***P*-value**
CPVI, *n (%)*	71 (100)	30 (100)	1.000	5 (45.4)	3 (100)	0.718
Mitral isthmus line, *n (%)*	6 (8.5)	8 (26.7)	0.035	1 (9.1)	1 (33.3)	0.528
Roof line, *n (%)*	12 (16.9)	12 (40)	0.013	2 (18.2)	1 (33.3)	0.889
Anterior line, *n (%)*	4 (5.6)	4 (13.3)	0.365	2 (18.2)	0 (0)	0.516
CFAE, *n (%)*	5 (7.0)	4 (13.3)	0.527	6 (54.5)	0 (0)	0.238
CTI, *n (%)*	2 (2.8)	2 (6.7)	0.728	0 (0)	0 (0)	1.000
SVC, *n (%)*	7 (9.9)	9 (30)	0.025	1 (9.1)	0 (0)	0.516

HFpEF, heart failure with preserved ejection fraction; HFmrEF, heart failure with mid-range ejection fraction; HFrEF, heart failure with reduced ejection fraction; CPVI, circumferential pulmonary vein isolation; CFAE, complex fractionated atrial electrogram; CTI, cavo-tricuspid isthmus; SVC, superior vena cava.

In 14 redo procedures, PV reconnections were observed in 5 patients (45.4%) in HFpEF group, which was comparable with those in HFmrHF + HFrEF group (100%, *p* = 0.718). There was no significant difference in the ablation of PV reisolation, mitral isthmus line, roof line, anterior wall line, CFAE, CTI, and SVC between the two groups.

No major peri-procedural complication was observed in both the initial and redo procedures.

### AF recurrence after AI-guided ablation procedures

**Figure 2:**
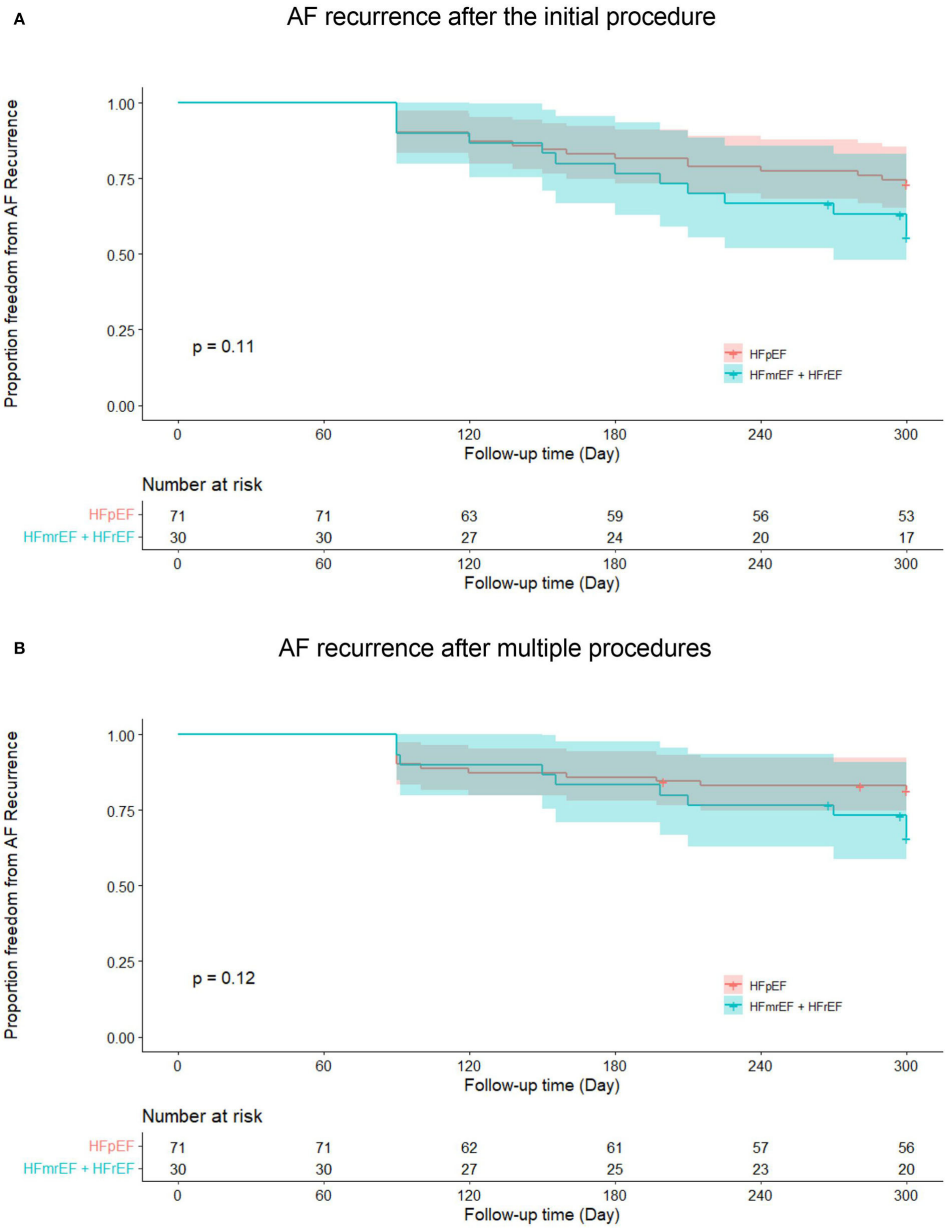
Kaplan–Meier curves show AF recurrence after the initial **(A)** and multiple procedures **(B)** in heart failure with preserved ejection fraction and heart failure with mid-ranged ejection fraction + heart failure with reduced ejection fraction groups. AF, atrial fibrillation; HFpEF, heart failure with preserved ejection fraction; HFmrEF, heart failure with mid-range ejection fraction + heart failure; HFrEF, heart failure with reduced ejection fraction.

In HFpEF group, 15 patients (21.1%) experienced recurrent AF, while 10 patients (33.3%) in HFmrEF + HFrEF group experienced AF recurrence after multiple procedures during a median follow-up of 32.0 (18.2, 37.6) months. Figure 2 shows the Kaplan–Meier curves for AF recurrence after the initial procedure (Log-rank *p* = 0.110) and multiple procedures (Log-rank *p* = 0.120) in both groups. In multivariable model, NYHA class III (*HR* 3.376, 95% *CI* 1.493–7.634, *p* = 0.003), non-paroxysmal AF (*HR* 3.314, 95% *CI* 1.050–10.463, *p* = 0.041) and early AF recurrence (*HR* 3.237, 95% *CI* 1.440–7.278, *p* = 0.004) were independently associated with AF recurrence after multiple procedures (Table 3).

**Table 3:** Univariate and multivariate analyses for risk factors of AF recurrence after multiple procedures.

**Variables**	**Univariate analysis**		**Multivariate analysis**	
	**HR (95% CI)**	***P*-value**	**HR (95% CI)**	***P*-value**
NYHA Class III	5.235 (2.359–11.618)	< 0.001	3.376 (1.493–7.634)	0.003
Non-paroxysmal AF	5.772 (1.974–16.880)	0.001	3.314 (1.050–10.463)	0.041
LAD ≥ 40mm	4.157 (1.243–13.899)	0.021	1.874 (0.506–6.940)	0.347
NT-ProBNP ≥ 800 pg/ml	3.583 (1.343–9.554)	0.011	1.677 (0.589–4.773)	0.332
Early AF recurrence	3.011 (1.361–6.661)	0.006	3.237 (1.440–7.278)	0.004

HR, hazard ratio; CI, confidence interval; NYHA, New York Heart Association; AF, atrial fibrillation; LAD, left atrial diameter; NT-ProBNP, N-terminal pro-B type natriuretic peptide.

### The composite endpoint

**Figure 3:**
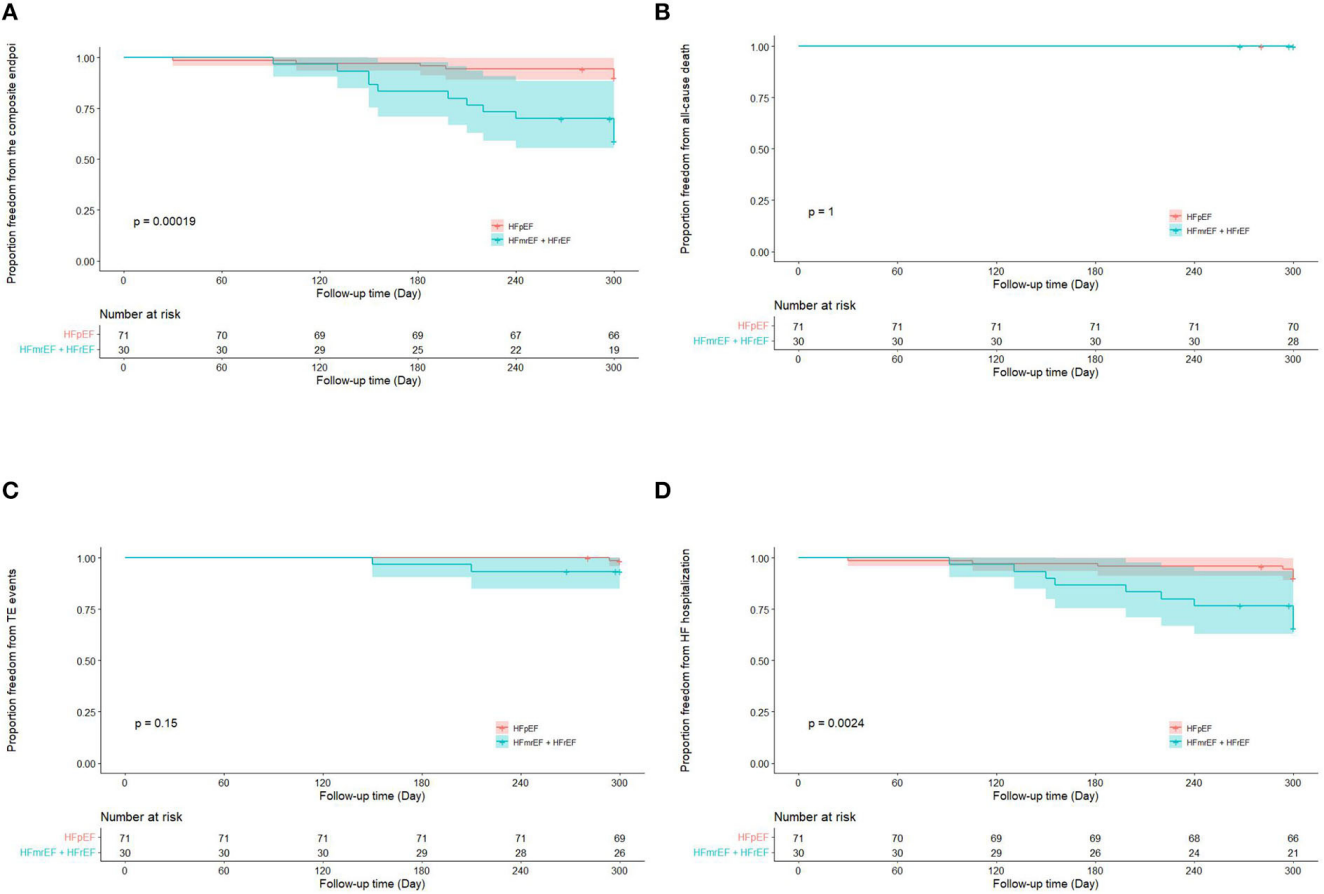
Kaplan–Meier curves showing the incidence of the composite endpoint **(A)**, all-cause death **(B)**, thromboembolic events **(C)**, heart failure hospitalization **(D)** in heart failure with preserved ejection fraction and heart failure with mid-ranged ejection fraction + heart failure with reduced ejection fraction groups. AF, atrial fibrillation; TE, thromboembolism; HF, heart failure; HFpEF, heart failure with preserved ejection fraction; HFmrEF, heart failure with mid-range ejection fraction + heart failure; HFrEF, heart failure with reduced ejection fraction.

**Figure 4:**
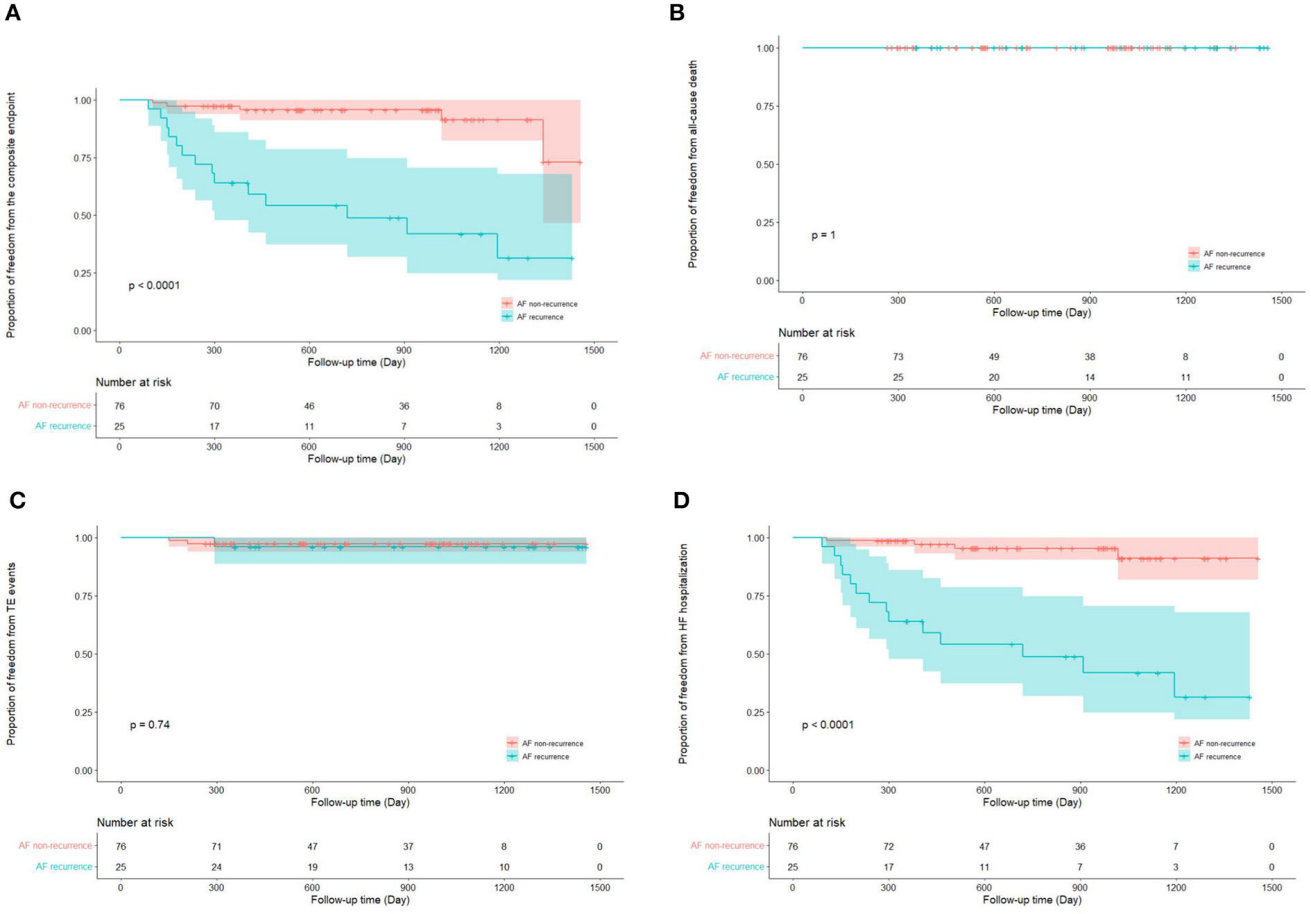
Kaplan–Meier curves show the incidence of the composite endpoint **(A)**. All-cause death **(B)**, thromboembolic events **(C)**, heart failure hospitalization **(D**) in patients with and without atrial fibrillation recurrence after multiple procedures. AF, atrial fibrillation; TE, thromboembolism; HF, heart failure.

Totally, the composite endpoint occurred in 19 (18.8%) patients, among whom 3 (15.8%) had TE events and 16 (84.2%) had HF hospitalization. No patient died during the follow-up period. Figure 3 shows the Kaplan–Meier curves for the cumulative incidence of the composite endpoint (Log-rank *p* < 0.001), all-cause death (Log-rank *p* = 1.000), TE events (Log-rank *p* = 0.150), and HF hospitalization (Log-rank *p* = 0.002) of HFpEF and HFmrEF + HFrEF groups. In addition, Figure 4 shows the Kaplan-Meier curves for the cumulative incidence of the composite endpoint (Log-rank *p* < 0.001), all-cause death (Log-rank *p* = 1.000), TE events (Logrank *p* = 0.740), and HF hospitalization (Log-rank *p* < 0.001) in patients with and without recurrent AF. In multivariable analysis, a history of hypertension (*HR* 4.667, 95% *CI* 1.433–15.203, *p* = 0.011), LVEF < 50% (*HR* 5.390, 95% *CI* 1.911–15.203, *p* = 0.001) and recurrent AF after multiple procedures (*HR* 7.542, 95% *CI* 2.355–24.148, *p* = 0.001) were independently associated with the presence of composite endpoint after adjusting for the confounding factors (Table 4).

**Table 4:** Univariate and multivariate analyses for risk factors of the composite endpoint.

**Variables**	**Univariate analysis**		**Multivariate analysis**	
	**HR (95% CI)**	***P*–value**	**HR (95% CI)**	***P*-value**
Non-paroxysmal AF	5.489 (1.592–18.919)	0.007	1.632 (0.403–6.605)	0.492
Hypertension	3.133 (1.039–9.451)	0.043	4.667 (1.433–15.203)	0.011
LAD ≥ 40 mm	1.082 (4.688–20.318)	0.039	2.525 (0.532–11.993)	0.244
LVEF < 50%	4.835 (1.912–12.223)	0.001	5.390 (1.911–15.203)	0.001
Recurrent AF after multiple procedures	10.471 (3.758–29.174)	< 0.001	7.542 (2.355–24.148)	0.001

HR, hazard ratio; CI, confidence interval; AF, atrial fibrillation; LAD, left atrial diameter; LVEF, left ventricular ejection fraction.

### The functional outcomes

**Figure 5:**
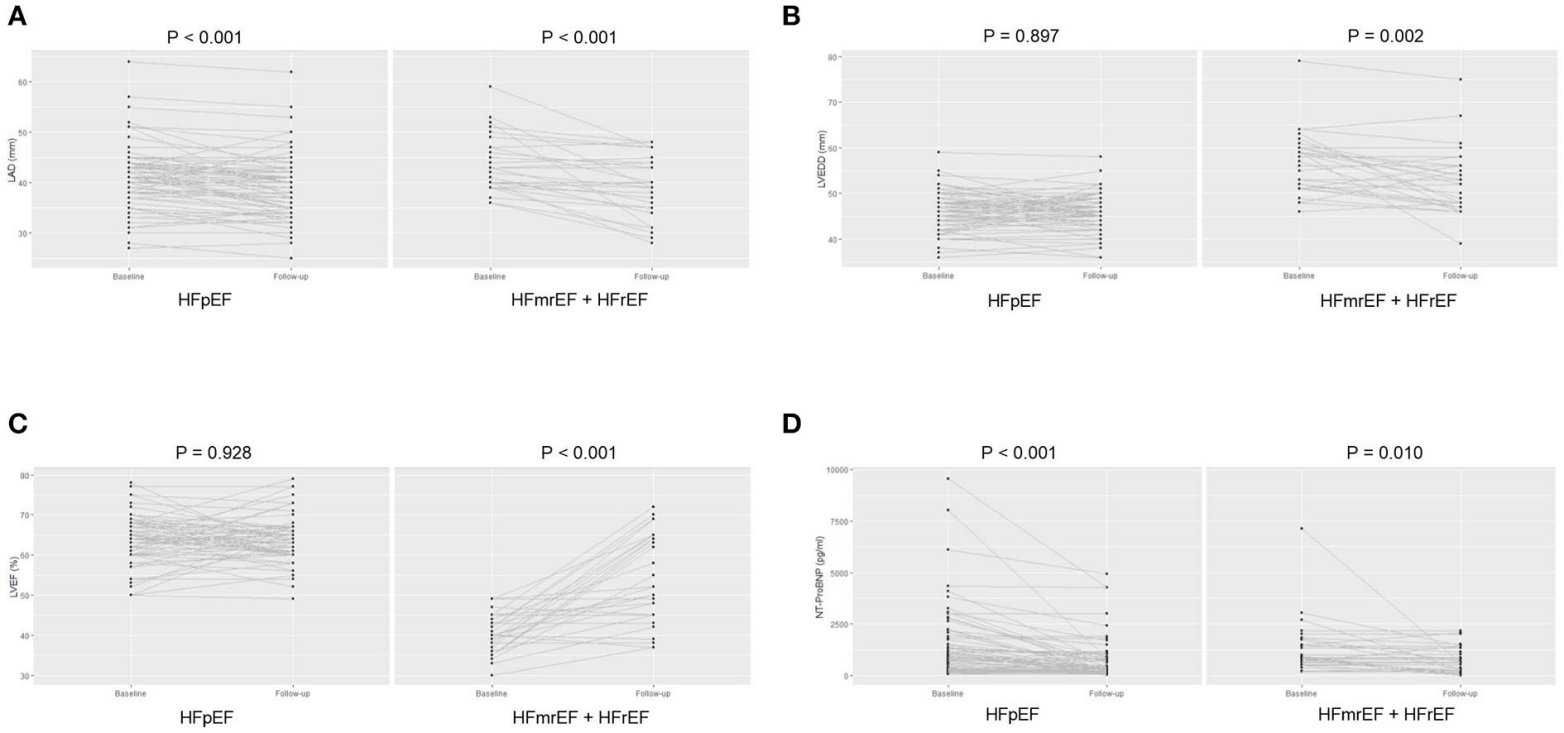
The left atrial diameter **(A)**, left ventricular end-diastolic diameter **(B)**, and left ventricular ejection fraction **(C)**, N-terminal pro-B type natriuretic peptide level **(D)** of patients at baseline and the end of follow-up in heart failure with preserved ejection fraction and heart failure with mid-ranged ejection fraction + heart failure with reduced ejection fraction groups. LAD, left atrial diameter; LVEDD, left ventricular end-diastolic diameter; LVEF, left ventricular ejection fraction; NT-ProBNP, N-terminal pro-B type natriuretic peptide; HFpEF, heart failure with preserved ejection fraction; HFmrEF, heart failure with mid-range ejection fraction + heart failure; HFrEF, heart failure with reduced ejection fraction.

Compared with baseline status, both groups showed an improved NYHA class [HFpEF: 2 (1, 2) *vs*. 2 (2, 2), paired *p* < 0.001; HFmrEF + HFrEF: 2 (1, 2) *vs*. 2 (2, 3), paired *p* = 0.016] at the end of follow-up. Regarding the TTE parameters, LAD significantly reduced (39.4 ± 6.4 *vs*. 41.1 ± 6.2, paired *p* = 0.001) at the end of follow-up, while LVEDD and LVEF were comparable between baseline and the end of follow-up in HFpEF group. However, LAD (39.0 ± 6.3 *vs*. 43.6 ± 5.6, paired *p* < 0.001) and LVEDD (52.5 ± 7.2 *vs*. 56.4 ± 6.7, paired *p* = 0.002) both significantly reduced, and LVEF significantly improved (53.5 ± 11.0 *vs*. 40.6 ± 5.0, paired *p* < 0.001) at the end of follow-up in HFmrEF + HFrEF group (Figures 5A–C). The NT-ProBNP level significantly reduced at the end of follow-up compared with baseline in both groups [HFpEF: 334.3 (187.1, 821.8) *vs*. 859.2 (308.4, 1903.0), paired *p* < 0.001; HFmrEF + HFrEF: 629.1 (262.2, 1364.0) *vs*. 871.5 (599.2, 1737.5), paired *p* = 0.010] (Figure 5D).

## Discussion

### Major findings

The major findings of the present study are as follows: (i) 75% of patients with AF and concomitant HF could achieve long-term sinus rhythm after AI-guided RFCA procedures, irrespective of LVEF subtypes. (ii) compared with patients with impaired LVEF, those with preserved LVEF were associated with a reduction in the composite endpoint of all-cause death, TE events and HF hospitalization. (iii) LVEF < 50% and recurrent AF after multiple procedures independently predicted the composite endpoint. (iv) AI-guided RFCA was associated with improved HF symptom and reverse remodeling in TTE in AF patients complicated with HF, regardless of LVEF.

### AI-guided RFCA in AF

Radiofrequency catheter ablation has been an increasingly established curative treatment for AF during the past decades. To achieve long-term success, the durable and transmural lesions created by RF energy are essential. Different modalities have been employed to facilitate the ablation procedure and improve the long-term ablation outcomes, among which, AI has been shown to serve as a novel marker associated with lesion transmurality and durability in RFCA procedures. Previous studies revealed that AI-guided ablation could improve the quality of scar formation (22) and increase the first-pass rate of PV isolation and roof line block (23, 24). Moreover, AI-guided ablation could result in a lower incidence of AT relapse post-blanking period (18–20, 25). However, the outcome of AI-guided ablation in AF and concomitant HF remains unclear. In the present study, an overall sinus rhythm maintenance rate of 75.2% post-multiprocedure was observed, which was lower than previous reports (18–20). We attribute this discrepancy to higher prevalence of non-paroxysmal AF, concomitant HF, larger mean LAD, and longer follow-up period of the present study.

### Ablation outcomes of AF and HF

The long-term success rate of RFCA in patients with AF and concomitant HF has been controversial. Depending on different characteristics of the study population, different ablation strategies, and different follow-up durations, the AF recurrence rate varied between 11 and 55% (26–28). The present study showed that the long-term success rate of single-procedure and multi-procedure was 65 and 75%, respectively.

In addition, debating results have been observed as for the impact of HF subtypes on the ablation outcomes. A registry study showed that AF recurrence was more often presented in patients with HFpEF (29). However, other studies found that the long-term AF recurrence rate was comparable between AF patients with preserved and impaired LVEF (30–32). A recent meta-analysis including 1,505 individuals showed a similar pooled efficacy of RFCA in HFpEF and HFrEF (33). The findings of the present study were in line with the previous results. This may be due to the similar pathophysiological processes and consequent atrial adverse remodeling in both HFpEF and HFrEF which hamper the efficacy of RFCA.

### Prognosis of AF and HF after RFCA

The prospective randomized multi-center CASTLE-AF trial (8) demonstrated a significant reduction in the composite endpoint in patients with AF and HFrEF who underwent RFCA procedures. A recent meta-analysis (34) also observed a better prognosis RFCA compared with rate control or medical rhythm control in AF patients complicated with HF. However, the impact of RFCA on the prognosis of AF patients with HFrEF and HFpEF remains controversial. Previous study showed that the prognosis of patients with AF and HFrEF is poorer that those with HFpEF (35). In contrast, two recent studies found that the incidence of adverse events and functional outcomes were comparable between AF patients with HFrEF and HFpEF (30, 32). These inconsistent results may be attributed to several factors, such as patient characteristics, HF etiology, follow-up duration, and ablation strategy. In addition, the improvement of prognosis in HF with impaired LVEF may be partially explained by the presence of tachycardia-induced cardiomyopathy, which is poorly recognized pre-ablation (36). However, it remains unclear of better patient selection and timing for RFCA in persistent AF with HF. A recent study (36) indicated that patients with normal to moderate LV dilation, resting heart rate ≥ 80 bpm and HFmrEF may be candidates for early RFCA to achieve LVEF normalization.

Although the overall outcomes seem to be improved with RFCA, it is noteworthy that this effect is predominated by individuals with favorable ablation outcome. Previous studies have revealed that AF recurrence post-ablation is an independent risk factor for the adverse events in both HFrEF (37) and HFpEF (28). In accordance with these observations, the present study found that the recurrent AF after multiple procedures is associated with 6-times higher risk of the composite endpoint. All these findings strongly underline the importance of sinus rhythm maintenance after RFCA procedures to improve the HF prognosis.

### Limitations

The present study has several limitations. Firstly, the study is a retrospective observational single-center study. We performed multivariate analysis to avoid the potential bias which might have been introduced by the imbalance of the baseline characteristics between the two study groups. Secondly, we combined the HFmrEF and HFrEF into one group due to the relatively small sample size. However, the clinical course and prognosis of the two different subtypes of HF are not identical, and future studies are warranted to further investigate the prognosis of these subgroups of patients. Thirdly, a trend toward higher AF recurrence rate in HFmrEF + HFrEF group was detected although it did not reach the statistical significance. We cannot entirely preclude the possibility of insufficient power result from the relatively small sample size. To better address the issue, studies with larger cohort are needed. Fourthly, the patients in the study underwent 24-h Holter monitoring, rather than implanted loop recorder during follow-up, which may underestimate the recurrence rate. Fifthly, the higher composite endpoint rate in HFrEF group was mainly driven by the higher incidence of HF hospitalization, whereas the number of patients with other events (all-cause death and TE events) was very small. Hence, it remains to be validated whether HFrEF could truly predict the events.

## Conclusion

In conclusion, long-term success could be achieved in 75% of patients with AF and concomitant HF after AI-guided RFCA procedures, irrespective of LVEF subtypes. Compared with patients with impaired LVEF, those with preserved LVEF were associated with a reduction in the composite endpoint of all-cause death, TE events and HF hospitalization. LVEF < 50% and recurrent AF after multiple procedures independently predicted the incidence of the composite endpoint. AI-guided RFCA was associated with improved HF symptom and reverse remodeling in TTE in AF patients complicated with HF, regardless of LVEF subtypes. Further large-cohort studies are warranted to confirm these observations.

## Data availability statement

The original contributions presented in the study are included in the article/supplementary material, further inquiries can be directed to the corresponding author.

## Ethics statement

The studies involving human participants were reviewed and approved by the Ethics Committee of Fuwai Yunnan Cardiovascular Hospital. The patients/participants provided their written informed consent to participate in this study.

## Author contributions

YQ and GN conceptualized the idea. YQ, ZZ, and XC organized the database and performed literature search and wrote the first draft of the manuscript. YQ and XC performed the statistical analysis. YG, MF, KL, JG, and TG critically reviewed the manuscript. All authors contributed to the manuscript and approved the final version.

## Funding

This study was supported by the Fuwai Yunnan Cardiovascular Hospital, Yunnan Provincial Cardiovascular Disease Clinical Medical Center Project (No. FZX2019-06-01).

## Conflict of interest

The authors declare that the research was conducted in the absence of any commercial or financial relationships that could be construed as a potential conflict of interest.

## Publisher's note

All claims expressed in this article are solely those of the authors and do not necessarily represent those of their affiliated organizations, or those of the publisher, the editors and the reviewers. Any product that may be evaluated in this article, or claim that may be made by its manufacturer, is not guaranteed or endorsed by the publisher.
